# Kardiale stereotaktische Strahlentherapie induziert eine Umprogrammierung des elektrischen Reizleitungssystems

**DOI:** 10.1007/s00066-021-01891-1

**Published:** 2021-12-20

**Authors:** Oliver Blanck, Judit Boda-Heggemann, Stephan Hohmann, Felix Mehrhof, David Krug

**Affiliations:** 1 Klinik für Strahlentherapie, Universitätsklinikum Schleswig-Holstein, Campus Kiel, Kiel, Deutschland; 2 Klinik für Strahlentherapie und Radioonkologie, Universitätsmedizin Mannheim, Medizinische Fakultät Mannheim, Universität Heidelberg, Mannheim, Deutschland; 3 Klinik für Kardiologie und Angiologie, Herzrhythmus Centrum, Medizinische Hochschule Hannover, Hannover, Deutschland; 4 Klinik für Radioonkologie und Strahlentherapie, Charité – Universitätsmedizin Berlin, Berlin, Deutschland

## Hintergrund

Die kardiale stereotaktische Strahlentherapie (cSBRT), auch Herzradiochirurgie oder Radioablation (RA) genannt, hat in der Behandlung von Patienten mit refraktären ventrikulären Tachykardien (VT) einen deutlichen therapeutischen Effekt im Sinne einer Reduktion der VT-Ereignisse und assoziierter Auslösungen der Implantierbarer-Kardioverter-Defibrillator(ICD)-Interventionen (Überstimulation, Schock) erzielt. Der für die cSBRT bislang postulierte Wirkmechanismus der radiogenen Fibrose erklärt jedoch nicht die Geschwindigkeit und das klinische Ausmaß der VT-Reduktionen nach cSBRT.

## Material und Methoden

In der Arbeit von Zhang et al. [1] wurden zunächst im Rahmen von Autopsien die Herzen von vier verstorbenen Patienten nach cSBRT im Rahmen einer Phase-I/II-Studie histopathologisch auf das Vorhandensein einer Fibrose im Zielgebiet untersucht. Anschließend wurden in einem Mausmodell die Effekte einer cSBRT mit 25 Gy histopathologisch, molekularbiologisch und elektrophysiologisch untersucht. Ergänzend wurden diese Experimente an Mäusen mit einem induzierten Myokardinfarkt sowie an Mäusen mit induzierter Überexpression oder funktionellem Knock-out des Notch-Signalwegs wiederholt. Schließlich wurden Untersuchungen bezüglich Proteinexpression an im Rahmen von Herztransplantationen explantierten Herzen von Patienten mit oder ohne cSBRT durchgeführt.

## Ergebnisse

An den posthum untersuchten Herzen der cSBRT-Patienten ließ sich trotz eines deutlichen klinischen Effekts der cSBRT keine signifikante Fibrose in den bestrahlten Arealen nachweisen. Dies wurde auch im Mausmodell nach cSBRT bestätigt. Die elektrophysiologische Beurteilung von mit 25 Gy bestrahlten Mäuseherzen zeigt einen supraphysiologischen elektrischen Phänotyp mit einer beschleunigten Leitungsgeschwindigkeit und einem verkürzten QRS-Komplex im EKG. Dies wurde auf eine Erhöhung der Expression von Na_V_1.5-Ionenkanälen und Cx43-Gap-Junction-Proteinen zurückgeführt. Durch gezielte Induktion bzw. Herunterregulierung wurde der Notch-Signalweg als Mechanismus zur Hochregulation der Na_V_1.5-Expression nach cSBRT identifiziert. Die Erhöhungen der Na_V_1.5- und Cx43-Expression waren anhaltend. Die elektrophysiologischen Effekte im Sinne der beschleunigten Reizleitung und Verkürzung des QRS-Komplexes ließen sich interessanterweise im Mausmodell auch bei niedrigeren Einzeitdosen von 15 bzw. 20 Gy beobachten. Die Effekte auf Proteinexpression und Elektrophysiologie waren bei Bestrahlung nach experimentell induziertem Myokardinfarkt auf das funktionsfähige Myokard beschränkt und ließen sich nicht im Narbengewebe nachweisen. Die vermehrte Na_V_1.5-Expression ließ sich über zwei Jahre nach cSBRT in der Zielregion bei einem im Rahmen einer Herztransplantation explantierten Herzen nachweisen. Eine Auswertung der EKG-Daten von Patienten aus einer klinischen Phase-I/II-Studie zeigte eine Verkürzung des QRS-Komplexes nach cSBRT bei 13 von 19 Patienten, während es bei 5 Patienten zu einer Verlängerung kam.

## Schlussfolgerung der Autoren

Diese Arbeit liefert Hinweise für eine strahleninduzierte Umprogrammierung der kardialen Reizleitung als zugrunde liegenden Wirkmechanismus einer cSBRT mit 25 Gy.

## Kommentar

Die Arbeit von Zhang et al. [1] ist bahnbrechend und richtungsweisend für die strahlentherapeutische Behandlung von Herzrhythmusstörungen und für das Verständnis der Wirkmechanismen von hohen Einzeldosen am Herzen. Die ursprüngliche Idee der gezielten Modulation der kardialen Reizleitung durch Induktion einer radiogenen Fibrose infolge einer ablativen Radiotherapie wurde erstmals von Sharma et al. im Tiermodell präsentiert [2]. In dieser Arbeit konnten die Induktion einer myokardialen Fibrose und resultierende Reizleitungsblockierungen durch hohe Einzeitdosen von 40 bis 60 Gy nachgewiesen werden. Diese fibrotischen und elektrophysiologischen Effekte setzten ca. 6–8 Wochen nach Bestrahlung ein. Die These, dass eine Fibrosierung auch bereits nach 25 Gy entstehen würde, konnte kurze Zeit später widerlegt werden [3]. Eine transmurale radiogene Fibrose ließ sich im Tiermodell nur mit Einzeitdosen deutlich über 30 Gy induzieren. Trotzdem wurde für die ersten Behandlungen von Patienten mit refraktären ventrikulären Tachykardien (VT) eine kardiale stereotaktische Strahlentherapie (cSBRT) mit einer Einzeitdosis von 25 Gy verwendet und, wie sich schnell herausstellte, mit großem Erfolg [4–6].

Die erste größere Patientenserie mit 5 Patienten [4] und die darauffolgende ENCORE-VT-Studie mit 19 Patienten [5] der Arbeitsgruppe aus St. Louis zeigten eindrucksvoll, dass eine cSBRT des VT-auslösenden Substrats mit 25 Gy eine drastische Reduktion der VT und der resultierenden ICD-Interventionen (Überstimulation, Schock) um mehr als 80 % bewirken kann. Es war jedoch relativ schnell klar, dass eine radiogene Fibrose als zugrunde liegender Mechanismus vor dem Hintergrund des Ausmaßes und vor allem der Geschwindigkeit der VT-Reduktionen nach cSBRT, oft bereits nach wenigen Tagen, nicht plausibel ist [3, 7]. In Obduktionen von mit cSBRT behandelten Patienten konnte passend hierzu keine ausgeprägte lokale Fibrose im Zielgebiet nachgewiesen werden [1, 4, 8]. Weiterhin zeigte sich nach cSBRT bei den meisten Patienten eine stabile, teils sogar verbesserte kardiale Auswurfleistung [5].

In der Studie von Zhang et al. [1] zeigte sich nun in Untersuchungen im Mäusemodell, dass eine Einzeitdosis zwischen 20 und 25 Gy die Proteinexpression von Na_V_1.5-Ionenkanälen und Cx43-Gap-Junction-Proteinen im Herzen hochreguliert. Eine Hochregulation dieser Proteine hat zur Folge, dass die Reizleitung im bestrahlten Gebiet bereits nach wenigen Tagen deutlich beschleunigt wird. Der Effekt der cSBRT scheint also anders als der Effekt der invasiven Katheterablation nicht auf einem zytostatischen oder zytotoxischen Effekt, sondern vielmehr auf einer Modulation der myokardialen Reizleitung zu beruhen. Dieser pathophysiologische und strukturelle Unterschied zwischen Katheterablation und cSBRT konnte an einem explantierten Herzen eines mit beiden Verfahren behandelten Patienten demonstriert werden [1].

Aus diesen Erkenntnissen ergeben sich einige konkrete Fragen für die cSBRT. Eine betrifft die Nomenklatur. Vielfach wird die cSBRT auch als Radioablation bezeichnet, was vor dem Hintergrund des nun demonstrierten Mechanismus nicht mehr adäquat erscheint. Weiterhin stellt sich die Frage, ob möglicherweise auch Dosen < 25 Gy für den therapeutischen Effekt ausreichen können. Dosen unter 15 Gy schienen in der Arbeit von Zhang et al. [1] mit einer deutlich geringeren Effektivität einherzugehen. Aufgrund signifikanter Ungenauigkeiten in der cSBRT aufgrund komplexer Zielvolumenbestimmungen [9] und überlagernder Bewegungsmuster [10] scheint die bisher verwendete Dosis von 25 Gy derzeit weiterhin angemessen, bis eine deutliche Harmonisierung und Präzisierung der cSBRT erfolgt ist. Unklar ist weiterhin, wie lange die „Umprogrammierung“ anhält. Zhang et al. konnten im Tiermodell wie auch am explantierten Herzen zeigen, dass die Hochregulation der Proteine noch ein Jahr nach cSBRT anhält. Es stellt sich dennoch die Frage, ob für das Erreichen eines langfristigen Therapieerfolgs die Induktion einer radiogenen Fibrose vorteilhaft sein könnte, zum Beispiel durch die Verwendung inhomogener Dosisverteilungen im Zielgebiet.

## Fazit

Für eine Weiterentwicklung dieses innovativen Verfahrens sind klinische Studien (z. B. RAVENTA, NCT03867747 [10]) und eine Zusammenarbeit im Rahmen internationaler Großprojekte (z. B. STOPSTORM, www.stopstorm.eu) zur detaillierten Analyse erfolgter Behandlungen und der klinischen Ergebnisse elementar. Die Behandlungen sollten sich an etablierten und publizierten Standards bzgl. Patientenselektion, Therapie, Nachsorge und Dokumentation orientieren [11, 12].

*Oliver Blanck, Judit Boda-Heggemann, Stephan Hohmann, Felix Mehrhof, David Krug, Kiel, Mannheim und Heidelberg*
